# SARS-CoV-2 amyloid, is COVID-19-exacerbated dementia an amyloid disorder in the making?

**DOI:** 10.3389/frdem.2023.1233340

**Published:** 2023-07-06

**Authors:** Nathaniel G. N. Milton

**Affiliations:** School of Health & Life Science, Leeds Trinity University, Leeds, United Kingdom

**Keywords:** SARS-CoV-2, COVID-19, amyloid, dementia, Alzheimer's, amyloid-ß

## 1. Introduction

The COVID-19 pandemic, caused by infection with the SARS-CoV-2 virus, is associated with patients suffering neurological symptoms including cognitive impairment (Hosp et al., 2021; Spudich and Nath, 2022; Taquet et al., 2022) plus an increase in the levels of dementia and Alzheimer's disease (AD) progression (Chen et al., 2022; Gordon et al., 2022; Golzari-Sorkheh et al., 2023; Olivera et al., 2023). The SARS-CoV-2 genome and proteome have been sequenced and components of the virus targeted for diagnosis and therapy (Hu et al., 2021; Kukar et al., 2021; Mishra et al., 2021; Yadav et al., 2021). The SARS-CoV-2 spike protein forms a trimer (Wrapp et al., 2020) that binds the angiotensin-converting enzyme 2 (ACE2) receptor on host cell surfaces to gain entry into the cells (Benton et al., 2020). Mutations of the receptor binding domain of the SARS-CoV-2 spike protein are found in a number of the variants of concern that show altered binding to the ACE2 receptor (Kim et al., 2023) and this also influences neurological symptoms (Taquet et al., 2022). The SARS-CoV-2 virus has been found in post-mortem brains (Crunfli et al., 2022; Stein et al., 2022), but some studies have failed to detect it (Khan et al., 2022). The mechanism of SARS-CoV-2 entry into the brain may involve either transmission via the nasal cavity (Butowt and von Bartheld, 2022) and/or an impaired blood-brain barrier (Yang et al., 2022). The key question is if the SARS-CoV-2 virus itself causes dementia and/or if SARS-CoV-2 infection exacerbates existing dementia (Paterson et al., 2020; Danics et al., 2021; Ecarnot et al., 2023). Dementia has many forms (Garcia-Ptacek et al., 2014) and some forms are associated with infections (Shinjyo et al., 2021).

The human body contains a range of amyloid proteins which aggregate to form fibrils that are deposited as part of disease processes (Chiti and Dobson, 2017). A classical feature of AD is the deposition of amyloid plaques containing the amyloid-ß (Aß) protein (Rijal Upadhaya et al., 2014). These AD amyloid plaques can also contain other amyloid proteins including islet amyloid polypeptide (IAPP), which is linked to diabetes mellitus (Jackson et al., 2013; Young et al., 2015). Intracellular amyloid deposition is thought to trigger neurodegeneration and extracellular plaque formation (Oddo et al., 2006). The Aß peptide is neuroprotective, antiviral plus antibacterial (Pearson and Peers, 2006; Bourgade et al., 2016; Huang, 2023) and Aß may be activated in infectious disease states as a protective measure which may explain the suggested viral involvement in Aß aggregation plus AD (Ezzat et al., 2019; Khokale et al., 2020; Niklasson et al., 2020; Liu et al., 2023).

The SARS-CoV-2 virus proteome contains a number of amyloid-forming protein sequences (Charnley et al., 2022; Nystrom and Hammarstrom, 2022; Tayeb-Fligelman et al., 2023), raising the possibility that COVID-19-exacerbated dementia is either a SARS-CoV-2 amyloid disorder or that the SARS-CoV-2 amyloids are a trigger for deposition of another amyloid, such as Aß, resulting in onset or worsening of preexisting dementias such as AD. For the purposes of this opinion article, the focus will be the SARS-CoV-2 amyloid proteins plus amyloid Aß associated with AD and their potential involvement in COVID-19-exacerbated dementias.

## 2. SARS-CoV-2 amyloid plus interactions with endogenous amyloid

A range of viral and bacterial proteins have been shown to have amyloid properties and this has been suggested to play a role in the disease processes associated with the viruses and bacteria concerned (Evans et al., 2018; Ezzat et al., 2019; Leger et al., 2020; Sampson et al., 2020; Saumya et al., 2021).

The four main structural proteins of the SARS-CoV-2 virus are the spike, envelope, membrane, and nucleocapsid proteins (Yadav et al., 2021). The SARS-CoV-2 proteome also includes non-structural and accessory proteins that are synthesized in infected cells and contribute to virus assembly in the host (Crooke et al., 2020). Amyloid proteins identified in SARS-CoV-2 include the structural spike (Nystrom and Hammarstrom, 2022) and nucleocapsid (Tayeb-Fligelman et al., 2023) proteins plus the accessory proteins ORF6 and ORF10 (Charnley et al., 2022). Aggregation and amyloid fibril formation is a storage mechanism for peptide hormones synthesized in large quantities and then stored intracellularly prior to release when cells are activated (Nespovitaya et al., 2016). SARS-CoV-2 infected cells make multiple copies of the virus and could contain sufficient SARS-CoV-2 amyloid proteins to allow the generation of intracellular amyloid (Abavisani et al., 2022). However, the levels of SARS-CoV-2 found in neuronal tissues at post-mortem are often low and do not always correlate with pathological changes (Thakur et al., 2021). The SARS-CoV-2 amyloid (Charnley et al., 2022; Nystrom and Hammarstrom, 2022; Tayeb-Fligelman et al., 2023) could also be released from infected cells and contribute to extracellular amyloid deposits. Aggregation conditions plus post-translational modifications of amyloid proteins such as Aß can influence both the fibril structures and their interactions with other human proteins (Milton and Harris, 2009) and such interactions between SARS-CoV-2 amyloid proteins could have effects on neuronal function. Many of the amyloid fibrils associated with disease states are toxic in their aggregated state (Almeida and Brito, 2020) and the neurotoxicity of SARS-CoV-2 amyloid (Charnley et al., 2022) could contribute to the pathophysiology of the infection.

The SARS-CoV virus (Zhong et al., 2003) is related to the SARS-CoV-2 virus and enters cells via the same ACE2 receptor (Xu et al., 2020). The envelope protein of SARS-CoV contains an amyloid-forming region (Ghosh et al., 2015), which is identical to a sequence in the SARS-CoV-2 envelope protein, suggesting that SARS-CoV-2 envelope protein could also form amyloid. The interaction of SARS-CoV envelope amyloid with IAPP (Ghosh et al., 2015), an identical SARS-CoV-2 envelope protein region and the presence of IAPP in amyloid plaques associated with AD (Jackson et al., 2013) raises the possibility of an interaction between the SARS-CoV-2 envelope protein and amyloid plaques in AD. The SARS-CoV membrane protein also aggregates (Lee et al., 2005) and shows 90% homology with the SARS-CoV-2 membrane protein (Thomas, 2020), raising the possibility of an aggregating form of the SARS-CoV-2 membrane protein.

The spike protein of SARS-CoV-2 contains amyloid sequences but does not itself form amyloid fibrils in the trimeric form (Nystrom and Hammarstrom, 2022). Coincubation in the presence of proteases results in the formation of amyloid fibrils raising the possibility of *in vivo* SARS-CoV-2 spike protein degradation by endogenous proteases leading to amyloid formation. The SARS-CoV-2 spike protein also binds the Aß amyloid protein found in AD plaques (Idrees and Kumar, 2021). Complexes involving more than one amyloid-forming compound may play a role *in vivo* leading to toxic fibril forms (Young et al., 2015) and this raises the possibility that copolymers involving SARS-CoV-2 spike protein amyloid and Aß (Idrees and Kumar, 2021) could play a role in triggering AD-like pathology. The SARS-CoV-2 spike protein is itself neurotoxic and activates Aß expression (Kyriakopoulos et al., 2022) providing a clear link between these two amyloid-forming proteins and neurotoxicity.

The SARS-CoV-2 nucleocapsid protein is a viral RNA-binding protein and can form amyloid fibrils (Tayeb-Fligelman et al., 2023). Unlike the SARS-CoV-2 spike protein, the full-length nucleocapsid protein can form fibrillar structures (Tayeb-Fligelman et al., 2023). Amyloid formation by the SARS-CoV-2 nucleocapsid protein fragments occurs in the presence or absence of RNA (Tayeb-Fligelman et al., 2023). Amyloid-binding peptides have been suggested as therapeutic agents (Milton et al., 2012) and targeting the SARS-CoV-2 nucleocapsid amyloid regions with binding peptides is antiviral with the potential for development as a COVID-19 therapy (Tayeb-Fligelman et al., 2023).

**Figure 1:**
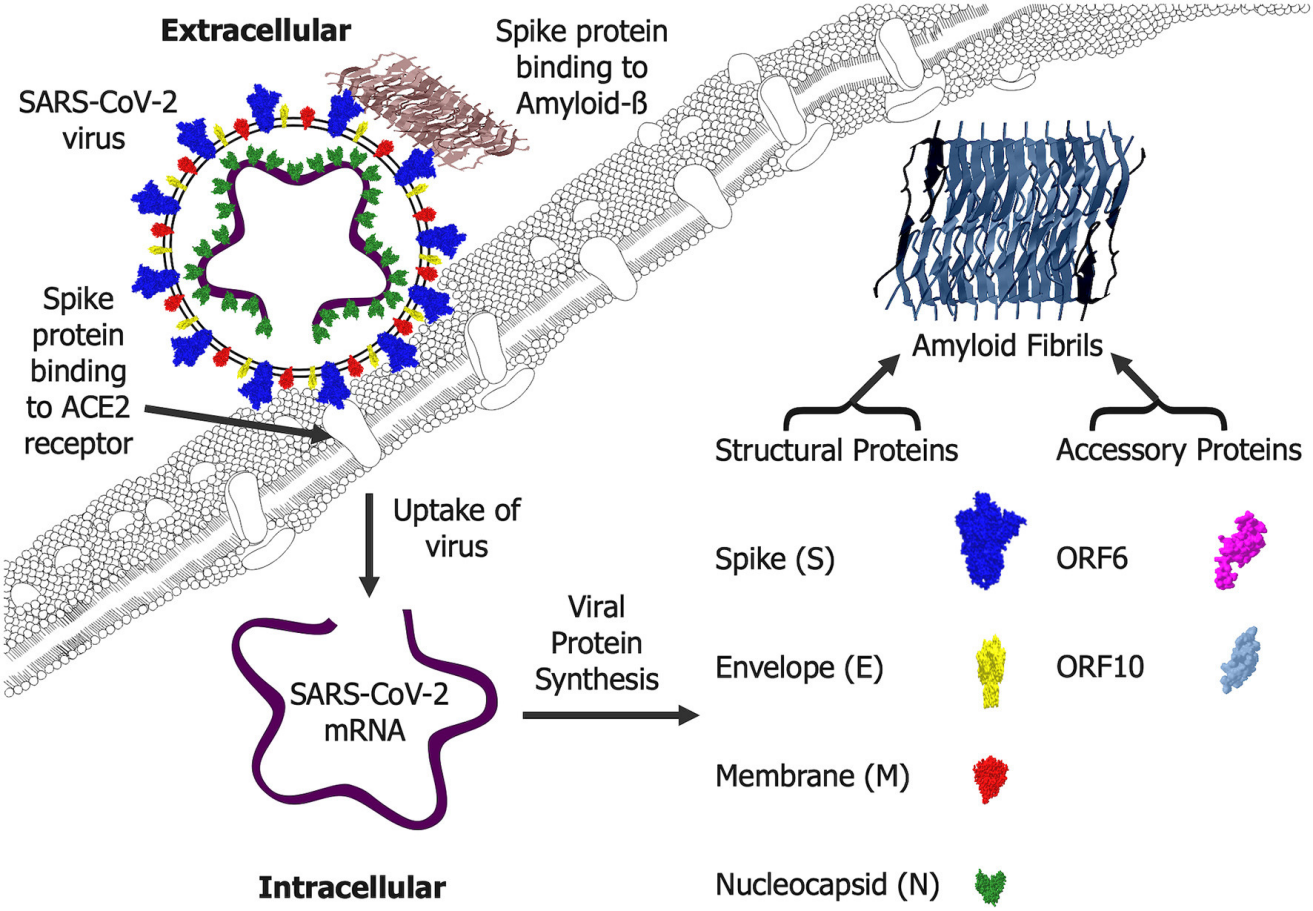
SARS-CoV-2 amyloid formation and interactions. Extracellular SARS-CoV-2 virus enters cells via an interaction of the spike protein (S) with the cellular angiotensin-converting enzyme 2 (ACE2) receptor. Once inside cells, the mRNA is translated into a range of viral proteins including the structural spike (S), envelope (E), membrane (M) and nucleocapsid (N) proteins plus the accessory proteins ORF6 and ORF10, all of which can form amyloid fibrils that are likely to be generated intracellularly. The spike (S) protein can also bind Amyloid-ß and this is likely to occur where the Amyloid-ß fibrils are deposited extracellularly.

The SARS-CoV-2 accessory proteins encoded by ORF6 plus ORF10 (Hassan et al., 2022a) form amyloid fibrils which are toxic to neurons (Charnley et al., 2022). Neurotoxic ORF6 amyloid fibrils (Charnley et al., 2022) could be generated in cells and may explain the observed toxicity when ORF6 is overexpressed (Lee et al., 2021). The presence of SARS-CoV-2 in the infected brain tissue (Douaud et al., 2022; Stein et al., 2022) raises the possibility that these neurotoxic forms could be generated in infected cells and cause some of the neurodegenerative changes observed. The SARS-CoV-2 infection may also target non-neuronal cells which express the ACE2 receptor in the olfactory bulb (Brann et al., 2020). The ORF10 protein has multiple roles linked to mitochondrial function and protein ubiquitination (Hassan et al., 2022b). Models of AD in which Aß is overexpressed show changes in mitochondrial function and protein ubiquitination (Aso et al., 2012) raising the possibility that the neurotoxic ORF10 amyloid protein (Charnley et al., 2022) could mimic Aß. The potential of SARS-CoV-2 amyloid proteins and their interactions with Aß is illustrated in Figure 1.

## 3. SARS-CoV-2 involvement in dementia

COVID-19 is primarily a respiratory disease (Li et al., 2020; Wu et al., 2020) and as such could indirectly affect the brain by causing hypoxic changes (Solomon et al., 2020; Thakur et al., 2021; Adingupu et al., 2023; Balsak et al., 2023). Hypoxia has been linked to dementia and neuropathological changes (Raz et al., 2016; Shobatake et al., 2022). Hypoxia is also linked to increased Aß generation (Pearson and Peers, 2006; Salminen et al., 2017) and SARS-CoV-2 infection is associated with hypoxia (Priemer et al., 2022) raising the possibility that these changes are not via a direct action of the virus within the brain. As such the COVID-19 exacerbated dementia could be a result of the hypoxia and other indirect changes rather than the SARS-CoV-2 virus entering the brain.

Memory deficits have also been suggested in patients with COVID-19 and the virus is associated with structural changes in the brain (Douaud et al., 2022; Serrano-Castro et al., 2022). Many amyloid proteins are linked to dementia (Garcia-Ptacek et al., 2014), and deposits of Aß are found in brain regions linked to memory and dementia (Furcila et al., 2018) raising the possibility that the SARS-CoV-2 amyloid proteins (Charnley et al., 2022; Nystrom and Hammarstrom, 2022; Tayeb-Fligelman et al., 2023) could play a role in COVID-19-exacerbated dementia. Changes in Aß levels, processing and deposition in amyloid plaques are also associated with SARS-CoV-2 infections (Ma et al., 2022; Priemer et al., 2022; Ziff et al., 2022).

The SARS-CoV-2 virus has been suggested to increase the toxicity of Aß (Chiricosta et al., 2021) and the spike protein binding to Aß plus other amyloid proteins (Idrees and Kumar, 2021) could promote aggregation and provide a mechanism for the increased Aß toxicity, a process that is linked to aggregation (Aleksis et al., 2017). This could provide a mechanism for the increased progression of dementia in AD patients who also have COVID-19 infections (Gordon et al., 2022). The hippocampal region of the brain is central to memory function and is a major site of neurodegeneration in AD (Furcila et al., 2018). The ACE2 receptor for the SARS-CoV-2 virus spike protein is found in hippocampal neuronal precursors (Hernandez-Lopez et al., 2023) and shows reduced expression in the hippocampus of AD patients, (Cui et al., 2021). The ACE2 protein is also expressed in non-neuronal cells within the brain (Brann et al., 2020) and this could contribute to symptoms of neuronal dysfunction (Spudich and Nath, 2022; Taquet et al., 2022) due to disruption of these cells in COVID-19 infections.

The SARS-CoV-2 spike protein is the main target of vaccines against COVID-19 (Wiersinga et al., 2020; Yadav et al., 2021; Patel et al., 2022) and as such antibodies may be in circulation to neutralize the effects of this SARS-CoV-2 infection by binding the spike protein and blocking its interaction with the ACE2 receptor. From a dementia perspective vaccination against the SARS-CoV-2 spike protein may be protective (Ecarnot et al., 2023), reducing infiltration of the virus and/or amyloid fragments of the spike protein into the brain but may not be able to reduce levels of the virus or deposited SARS-CoV-2 amyloids in the brains of patients already infected with the SARS-CoV-2 virus (Stein et al., 2022).

Persistent or Long COVID-19 is increasingly recognized as a condition (Perlis et al., 2022), is associated with old age and could be linked to SARS-CoV-2 amyloid (Charnley et al., 2022; Nystrom and Hammarstrom, 2022; Tayeb-Fligelman et al., 2023). The long-term persistence of symptoms beyond SARS-CoV-2 infection remains to be determined (Gordon et al., 2022; Serrano-Castro et al., 2022) and a post-pandemic change in dementia rates will take time to show. If COVID-19 is like other viral-induced dementias (Ezzat et al., 2019; Khokale et al., 2020; Leger et al., 2020; Niklasson et al., 2020; Saumya et al., 2021) and causes exacerbations of AD dementia (Liu et al., 2023; Olivera et al., 2023) it would be expected that an increase in AD levels post-pandemic may develop over time. The COVID-19 exacerbations of AD dementia (Liu et al., 2023; Olivera et al., 2023) may increase AD levels post-pandemic over time. It remains to be seen if the vaccination strategies (Patel et al., 2022) will protect from dementia. Changes in brain structure in patients who were scanned pre and post-COVID-19 infection suggest that the SARS-CoV-2 virus itself triggers changes (Douaud et al., 2022), future scans will determine whether this is long-lasting and linked to dementia.

## 4. Final considerations

A combination of discovered SARS-CoV-2 amyloid proteins (Charnley et al., 2022; Nystrom and Hammarstrom, 2022; Tayeb-Fligelman et al., 2023) and suggested possible amyloids (Lee et al., 2005; Ghosh et al., 2015) provides a potential source of amyloid in COVID-19 infections. As such infection with SARS-CoV-2 has the potential to result in the production of amyloid deposits within the host at both extracellular and intracellular locations. The ability of the SARS-CoV-2 virion to infect neuronal and non-neuronal cell types within the brain (Brann et al., 2020; Stein et al., 2022) suggests a potential for a contribution of these deposits to dementia (Taquet et al., 2022) in a manner like the endogenous amyloids associated with dementia (Garcia-Ptacek et al., 2014). The toxicity of some of the SARS-CoV-2 amyloid forms (Charnley et al., 2022) could directly cause brain changes (Douaud et al., 2022) and lead to COVID-19 exacerbated dementia (Hosp et al., 2021; Taquet et al., 2022). The interactions with endogenous Aß plus other amyloids (Idrees and Kumar, 2021) provide an alternative mechanism for SARS-CoV-2 amyloid proteins triggering the deposition of other forms of amyloid with consequent dementia such as AD (Chiti and Dobson, 2017). In conclusion, I propose that the SARS-CoV-2 amyloid proteins (Lee et al., 2005; Ghosh et al., 2015; Charnley et al., 2022; Nystrom and Hammarstrom, 2022; Tayeb-Fligelman et al., 2023) could play a potentially causative role in COVID-19 exacerbated dementia (Hosp et al., 2021; Taquet et al., 2022) and as such may be disease-modifying targets (Tayeb-Fligelman et al., 2023).

## Author contributions

NM wrote the manuscript and prepared the figure.
